# Visual symptom provocation in skin picking disorder: an fMRI study

**DOI:** 10.1007/s11682-017-9792-x

**Published:** 2018-01-05

**Authors:** Anne Schienle, Sonja Übel, Albert Wabnegger

**Affiliations:** Clinical Psychology, BioTechMedGraz, University of Graz, Universitätsplatz 2, 8010 Graz, Austria

## Abstract

Skin-picking disorder (SPD) is a common mental disorder characterized by recurrent and excessive picking of dermatological irregularities. Different disorder models have been developed to explain this behavior, but empirical evidence is still scarce. One model (the disgust-related disease avoidance model) suggests that SPD might be understood as pathological grooming elicited by skin imperfections that singal possible infection. Twenty-five women with SPD and 19 matched controls viewed and rated images depicting skin irregularities and smooth skin during functional magnetic resonance imaging. The participants did not engage in picking behavior. Relative to controls, SPD patients reported more disgust and urge to pick when looking at skin irregularities. This was accompanied by greater activation in the insula and amygdala, and stronger insula-putamen coupling. Disgust feelings elicited by viewing skin irregularities were positively correlated with activation of the insula and the putamen, in the clinical group. On personality questionnaires, the SPD patients reported elevated self-loathing and problems in regulating their disgust feelings. The current study provides first evidence for altered disgust processing in SPD patients.

## Introduction

Several mental disorders (e.g., borderline personality disorder, eating disorders) are associated with nonsuicidal self-injury, defined as the deliberate, self-inflicted destruction of body tissue (e.g., scratching, biting or cutting skin). Another often undiagnosed serious condition, skin-picking disorder (SPD), is characterized by recurrent and excessive picking of dermatological irregularities (e.g., smaller skin lesions, moles, pimples; American Psychiatric Association 2013). This repetitive manipulation (usually with the fingernails) causes severe skin damage and clinically significant distress or impairment in important areas of functioning (Odlaug and Grant 2008). In the DSM-5 (American Psychiatric Association 2013), SPD is listed in the section of obsessive–compulsive and related disorders (OCD). Symptoms of SPD are similar to those of OCD, since SPD patients pick their skin over and over again, often in response to recurrent thoughts and impulses. In these patients, the picking has the quality of a focused ritual and is typically experienced as being relieving. However, in some patients, skin picking occurs automatically and unconsciously (Walther et al. 2009). These patients report that they pick, and only much later notice that they have been picking (e.g., because of bleeding). Patients may present with a mixture of both focused and automatic picking styles (American Psychiatric Association 2013). SPD is a common mental disorder with reported prevalences ranging from 1.4 to 5.4% (American Psychiatric Association 2013; Grant et al. 2012).The clinical manifestation of SPD may occur across the life span, with the most common starting point being puberty. The disorder is more common in women than men, with a ratio of 3:1 (American Psychiatric Association 2013).

Disorder models (psychological, neurobiological) for explaining the origin as well as the maintenance of SPD are still incomplete. Some researchers have identified a high level of impulsivity, an enhanced emotion reactivity, or a reduced emotion regulation capacity in patients (Grant et al. 2012; Roberts et al. 2013; Snorrason et al. 2010).

Structural neuroimaging studies have found reduced integrity of white matter tracts connecting anterior cingulate cortices in SPD patients (Grant et al. 2013), a greater volume of the bilateral nucleus accumbens, and reduced cortical thickness in right frontal areas, compared to control participants and patients with trichotillomania (Roos et al. 2015). Since the nucleus accumbens is a key region of the brain reward system, and frontal areas are implicated in motor control, the anatomical changes might reflect reward deficiency and disinhibition of motor control.

To the best of our knowledge, there is only one functional magnetic resonance imaging (fMRI) study on SPD, conducted by Odlaug et al. (2016). SPD patients showed lowered activation in a cluster encompassing the striatum and frontal regions (e.g., anterior cingulate cortex) during the planning period of the Tower of London test, which assesses executive functioning. Neuroimaging studies, which have directly studied skin picking by means of a symptom provocation design, are still missing.

Interestingly, focused skin picking is elicited by visual cues, which are considered to be typical disgust elicitors. Dermatological irregularities reflecting skin disease elicit disgust in the majority of people (Schienle et al. 2002). Disgust researchers have offered different explanations as to why these skin conditions are disgust-relevant. One type of model explains disgust as an adaptive system that evolved to motivate disease-avoidance behavior (e.g., Curtis 2011; Davey 2011). It arose in our animal ancestors to facilitate detection of infectious risk and to drive hygienic behavior. Curtis (2011) distinguishes two basic modes of infection: ‘faecal–oral infections’ (e.g., by body secretions, contaminated food), and ‘skin contact infections’ (e.g., by micro- and macro-parasites). Both types of disease cues elicit health-protecting behaviors, such as rejection, distancing, and avoidance. Another kind of behaviors, which is especially relevant for skin-contact infection, includes grooming and cleaning aiming at the removal of pathogens. Self-grooming (e.g., in the form of scratching, picking) is a common behavioral strategy used by different species in order to reduce transmission of parasites (Prokop et al. 2014). This behavior can be elicited by tactile cues (e.g., itching) or visual cues. In a study by Prokop et al. (2014) students received visual-oral information on parasites in the form of a 45-minute lecture. This intervention (compared to a lecture on hormones) was able to activate self-grooming behavior (e.g., increased need for scratching, hand-washing). The degree of self-grooming in the students was positively correlated with their perceived own vulnerability to disease. Extending this idea, SPD might be conceptualized as a ‘grooming disorder’ (Grant and Stein 2014). The repeated picking and scratching of the patients’ own skin might be due to an oversensitive (disgust-driven) behavioral immune system that aims at the removal of potential pathogens from the skin (Schaller and Park 2011).

In the current fMRI investigation, we tested whether the presentation of images depicting minor skin irregularities would elicit disgust and urge to pick in SPD patients. We hypothesized that the presentation of disorder-relevant visual cues would be associated with activation of brain regions involved in disgust processing (insula) and motor preparation (e.g., motor cortex). Moreover, we investigated if SPD patients also report elevated trait disgust.

## Method

### Participants

Data from 25 women diagnosed with SPD according to DSM-5 (mean age: 35.9 years, SD = 15.33) and 19 healthy women (mean age: 33.8 years, SD = 13.2) were analyzed. Two patients had to be excluded due to excessive motion during scanning (> 1.2 mm). The two groups did not differ in age (p = .64) or years of education (M_patients_: 12.76, SD = 1.62; M_controls_: 13.11, SD = 2.21; p = .57). Exclusion criteria for the clinical sample were diagnoses of psychosis, substance abuse/ dependence and severe depressive symptoms. Any life-time diagnosis of a mental disorder led to exclusion from the control group.

All patients executed skin picking behavior with their fingernails, predominantly on their hands and arms. The reported average duration of picking was M = 156 min/ day (SD = 12.6). Clinical manifestation occurred in childhood or puberty (5–15 years of age) in 17 patients, whereas eight patients reported symptom onset in adulthood (> 18 years). The average age of onset was 16 years (SD = 9.6); the mean duration of symptoms was 19.5 years (SD = 12).

A board-certified clinical psychologist conducted a standardized clinical interview (Margraf 1994), which had been extended with SPD-related questions (e.g., onset, elicitors/ type of scratching). Diagnosed comorbidity included major depression (mild to moderate symptoms) in two patients, who received antidepressant medication (SSRI, SNRI). Participants were recruited by means of the outpatient clinic at the Department of Clinical Psychology (University of Graz, Austria) and by media advertisements. After participants were given a complete description of the study, written informed consent was obtained. The local ethics committee approved this study, which was carried out in accordance with the ethical principles established in the 2008 Declaration of Helsinki.

### Images and design

Thirty pictures of body parts (e.g. arms, legs, face) with minor skin irregularities (e.g., small skin lesions, pimples, moles), and 30 control images of the same body parts without irregularities were shown. The images were partly taken from a validated picture set for the elicitation of disgust feelings (Schienle et al. 2002) or were developed for the study. The participants viewed the images passively and did not engage in picking behavior. A camera system monitored adherence to the instruction. The pictures were presented in an event-related design in random order (à 2 s; inter-stimulus interval: 4–8 s). Five randomly selected images from the two conditions were rated by the participants regarding experienced disgust, tension and urge to pick, on 9-point Likert scales (1 = very low; 9 = very intense).

### Questionnaires

All participants answered the following questionnaires in an online survey prior to the MRI experiment:

- The Questionnaire for the Assessment of Disgust Proneness (QADP; Schienle et al. 2002) assesses the general tendency of a person to experience disgust across different situations (e.g., ‘You are just about to drink a glass of milk as you notice that it is spoiled’). The Cronbach’s alpha (total scale) was 0.92 in the present sample.
- The Scale for the Assessment of Disgust Sensitivity (SADS; Schienle et al. 2010) consists of seven items addressing difficulties to regulate one’s own feelings of disgust (e.g., ‘Experiencing disgust is stressful for me’). The Cronbach’s α of the scale was 0.95.
- The Questionnaire for the Assessment of Self-Disgust (QASD; Schienle et al. 2014) has two subscales “Disgusting Self” (nine items; Guttman’s λ4 = 0.81) and “Disgusting Ways” (six items; Guttman’s λ4 = 0.81) which assess disgust-related self-concept (e.g., “I find myself repulsive”) and behavior (e.g., “I regret my behavior”).
- The Skin Picking Scale revised (SPS-R; Gallinat et al. 2016) has two sub scales (4 items each) which describe “symptom severity” (Cronbach`s α = 0.96) and “impairment” (α = 0.95) by the picking during the last week. Each item is rated on 5-point scales (e.g., “How often to you feel the need to pluck or squeeze your skin?”).
- The Milwaukee Inventory for the Dimensions of Adult Skin-picking (MIDAS, Walther et al. 2009) has been constructed with a sample of SPD patients. Therefore, it was only answered by the clinical group of the current study. The MIDAS consists of a 6-item focused picking scale with a Cronbach’s α of 0.87 (‘I pick my skin when I am experiencing a negative emotion, such as stress, anger, frustration, or sadness’) and a 6-item automatic picking scale (‘I don’t notice that I have picked my skin until after it’s happened’; α = 0.80).

### MRI: recording and analysis

The MRI session was conducted with a 3T scanner (Skyra, Siemens, Erlangen, Germany) with a 32-channel head-coil. Functional runs were acquired using an echo-planar imaging protocol (number of slices: 35, descending, flip angle = 90°, slice thickness: 3 mm; matrix: 64 × 64 mm; TE = 30 ms; TR = 2290 ms; FoV: 192 mm; in-plane resolution = 3 × 3 × 3 mm); average duration = 11.28 min (SD = 1.33). Structural images were obtained using a T1-weighted MPRAGE sequence (voxel size: 0.9 × 0.9 × 0.9 mm; 192 transverse slices, FoV = 224 mm, slice thickness: 0.88 mm, TE = 1.89 ms, TR = 1680 ms; TI = 1000 ms, flip-angle = 8°). Field maps were obtained by using the following sequence: 35 slices, slice thickness: 3 mm, TR: 400 ms, short TE: 4.92 ms, long TE: 7.38, flip angle 60°, Fov: 192 mm, in-plane resolution = 3 × 3 × 3 mm with a magnitude and phase reconstruction. To increase the comfort for the participants and to decrease head movements, foam pads were used to stabilize participants’ heads. All analyses were conducted with SPM12 (version: 6906; Wellcome Department of Cognitive Neurology, London). For the analysis of functional data three volumes from the beginning of the time series were discarded to account for saturation effects.

In a first step motion correction was applied using realignment and unwarping with an additional field map that should correct additionally for possible field inhomogeneities. Afterwards acquisition timing was accounted during the slice timing step using the middle slice as reference scan. Subsequently, individuals T1-weighted images were segmented grey matter (GM), white matter (WM) and cerebrospinal fluid into the native space by using the tissue probability maps implemented in SPM 12 and a skull-stripped image was created. Segmentation used a light bias regularization, a bias FWHM of 60 mm, with light-clean up and an affine regularization to the ICBM space template for European brains. Slice-timed, realigned and unwarped images were matched to the skull-stripped image using the normalized mutual function. Afterwards obtained forward deformation fields during segmentation were used to bring functional images to MNI space. Finally, for smoothing a Gaussian kernel of 6 mm was applied.

We compiled vectors for each event of interest (picture onset) and entered them into the design matrix to model event-related responses by the canonical hemodynamic response function in the first level stage. Data were high pass filtered (128 s). Additionally, with the six movement parameters obtained during the realignment step we calculated individual motion patterns with the help of the motion fingerprint toolbox (Wilke 2012). The derived six motion fingerprint parameters were used as regressors of no interest. An AR(1) process was applied for prewhitening.

For the fMRI data, we computed planned t-contrasts (skin irregularities - smooth skin and smooth skin – skin irregularities) for within-groups and between-groups analyses. Additionally, age was considered as regressor of no interest for all analyses. We conducted exploratory whole-brain voxel intensity tests as well as region of interest (ROI) analyses for the insula, amygdala, the striatum and motor cortex (supplementary motor area, primary motor cortex) based on previous findings on SPD and OCD (Odlaug et al. 2016; Schienle et al. 2005).

Further, we conducted multiple regression analysis to correlate questionnaire scores and affective picture ratings with ROI activation. The used ROI masks were taken from the Harvard-Oxford cortical and subcortical structural atlases. Masks were created using the Wake Forest University (WFU) Pickatlas (Maldjian et al. 2003) and were forwarded to SPM´s ImCalc function to create masks with a probability threshold of 25%.

For all analyses, the height threshold was set at p < .005 uncorrected for at least 5 contiguous voxels. Results were small volume corrected and considered significant if the peak-level statistic was below p < .05 (corrected for family-wise error (FWE)).

We also conducted psychophysiological interaction (PPI) analyses (Friston et al. 1997) to investigate functional connectivity. PPI assesses the extent to which the experimental factor (contrast: skin irregularities - smooth skin) modulates the connectivity of a specific brain region (‘seed’) with other regions, in terms of condition-specific covariation in residuals. Subject-specific contrast images were entered into a two-sample t-test to compare groups. Age was considered as regressor of no interest. We defined the amygdala and the insula as seed regions, based on our fMRI findings on the group level. A 6 mm sphere was built around each significant peak based on the group analyses. For the PPI analyses the height threshold was set at p < .005 uncorrected for at least 5 contiguous voxels. Results were small volume corrected and considered significant if the peak-level statistic was below p < .05 (corrected for family-wise error (FWE)). The ROIs were the same as in the fMRI approach. The following specific hypotheses were tested:

- SPD patients show increased insula activation while viewing skin irregularities (contrast: skin irregularities – smooth skin) relative to controls.
- Experienced disgust while viewing skin irregularities (contrast: skin irregularities – smooth skin) positively correlates with insula activation in patients.

The following exploratory research questions were investigated (with correction for multiple testing):

c) SPD patients and controls differ in ROI activation (left/ right amygdala, left/ right striatum and left/right motor cortex) for the contrast skin irregularities - smooth skin; Bonferroni cut-off: alpha/6 = 0.008.
d) Correlations between ROI activation in both hemispheres (amygdala, insula, striatum, motor cortex) and state variables (urge to pick/ tension) as well as trait variables (e.g., SPS_R, MIDAS) were examined (Bonferroni cut-off for each scale: alpha/8 = 0.006).

## Results

### Self-report

Relative to the control group, the patients reported more skin-picking behavior (SPS_R), higher self-disgust (QASD) and disgust sensitivity (SADS). Results are depicted in Table 1. Patients’ scores for the two MIDAS sub scales were M_automatic picking_ = 19.74 (SD = 5.03) and M_focused picking_ = 21.68 (SD = 6.08).

**Table 1:** Self-report data

		Patients	Controls	t(p)
Questionnaires				
SPS-R:	total	18.36 (5.86)	1.16 (2.39)	13.30 (< 0.001)
	symptom severity	9.80 (2.35)	0.79 (1.51)	14.58 (< 0.001)
	impairment	8.56 (3.95)	0.37 (0.96)	9.99 (< 0.001)
QADP:	disgust proneness	2.45 (0.58)	2.24 (0.66)	1.10 (0.227)
SASD:	disgusting self	1.69 (0.94)	0.14 (0.24)	7.45 (< 0.001)
	disgusting ways	1.87 (0.98)	0.16 (0.23)	6.99 (< 0.001)
SADS:	disgust sensitivity	1.67 (1.18)	0.60 (0.50)	3.70 (< 0.001)
Image ratings [1..9]				
Skin irregularities:	disgust	6.03 (1.10)	4.51 (1.35)	4.12 (< 0.001)
	tension	5.73 (1.34)	2.72 (1.59)	6.81 (< 0.001)
	urge to pick	5.66 (1.75)	1.32 (0.50)	10.49 (< 0.001)
Smooth skin:	disgust	2.37 (1.00)	1.53 (0.92)	2.86 (0.007)
	tension	2.76 (1.19)	2.28 (1.82)	1.04 (0.303)
	urge to pick	2.89 (1.18)	1.09 (0.22)	6.49 (< 0.001)

*SPS-R* Skin Picking Scale revised, *QADP* Questionnaire for the Assessment of Disgust Proneness, *SASD* Scale for the Assessment of Self-Disgust, *SADS* Scale for the Assessment of Disgust Sensitivity

In order to compare the affective picture ratings (disgust, tension, urge to pick) for both image types (skin irregularities, smooth skin) between the groups (patients, controls), t-tests with Bonferroni correction (cut-off = 0.008) were computed. The patients rated skin irregularities as more disgusting, and experienced more tension and urge to pick than controls. When looking at smooth skin, the patients only reported greater urge to pick than controls (see Table 1). Within the patient group, skin irregularities elicited more disgust, tension and urge to pick than smooth skin (all ps < 0.01).

### fMRI

**Fig. 1:**
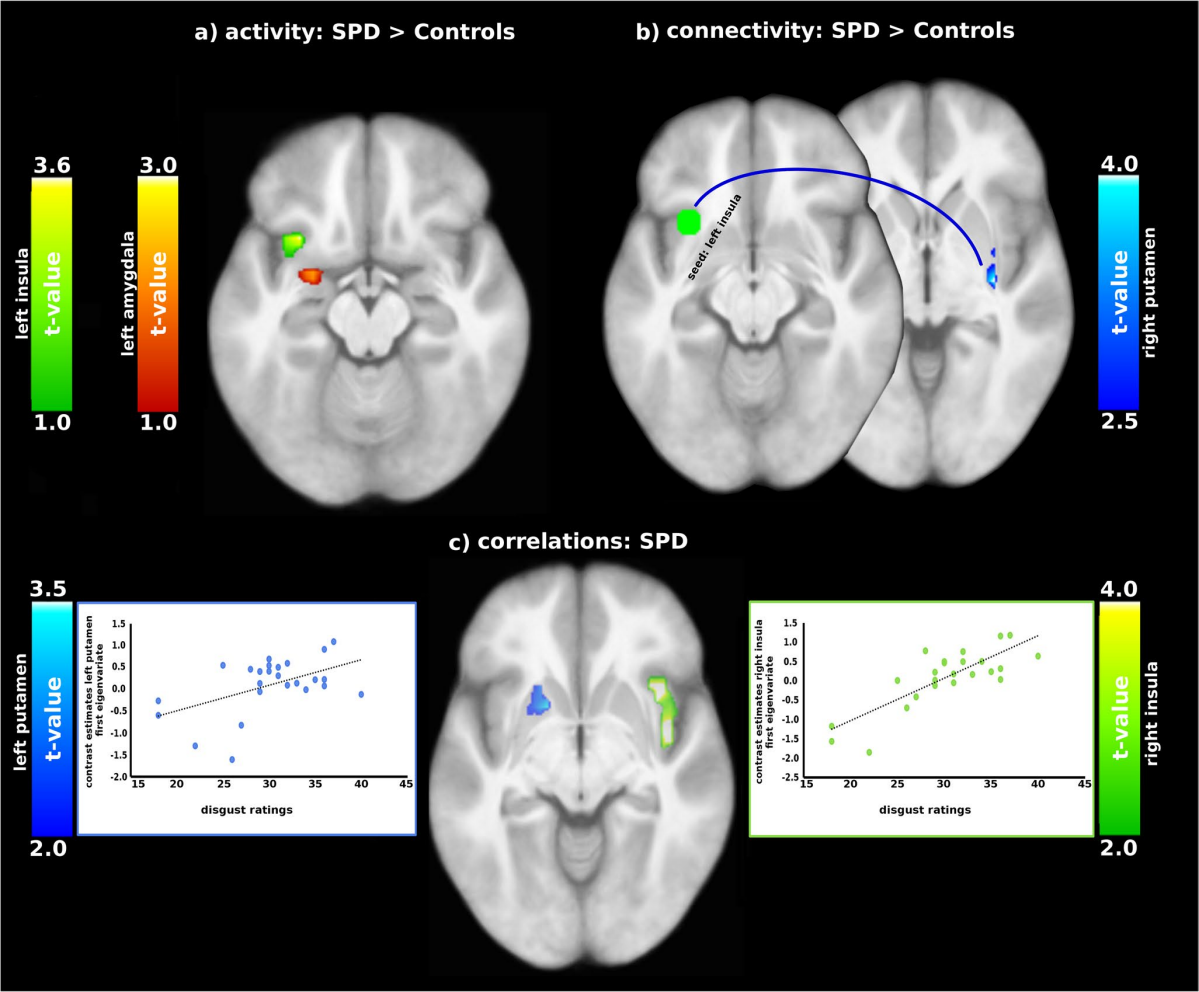
**a** Comparison of brain activity and **b** connectivity between SPD patients and controls; **c** correlations between disgust ratings and brain activity in SPD patients (contrast: skin irregularities - smooth skin)

Relative to controls, SPD patients showed greater activation in the left insula (MNI coordinate x,y,z: − 30,14, − 16, t = 3.63, p(FWE-corrected) = 0.049) and in the left amygdala (MNI coordinate x,y,z: − 27, − 1, − 22, t = 3.94, p(FWE-corrected) = 0.007 (contrast: patients - controls: skin irregularities - smooth skin; see Fig. 1).

### Correlation analyses

Simple regressions were computed between image ratings and ROI activation separately for patients and controls. Experienced disgust and tension correlated positively with activation of the insula and putamen for the contrast skin irregularities - smooth skin. (Table 2). None of the correlations for the control group reached statistical significance.

**Table 2:** Positive correlations between image ratings (skin irregularities) and ROI activation for patients

Region of interest	H	X	Y	Z	t	p(FWE)
Disgust						
Insula	R	45	− 7	− 7	8.45	<0.001^w^
Insula	L	− 42	− 10	− 4	4.94	0.007
Caudate nucleus	L	− 9	8	− 1	6.58	0.035^w^
Superior temporal gyrus	L	− 60	5	− 1	6.43	0.049^w^
Amygdala	L	− 27	− 1	− 16	3.63	0.029
Amygdala	R	30	− 10	− 16	3,68	0.028
Motor cortex	R	54	8	5	5.74	0.010
Putamen	L	− 15	14	− 7	4.08	0.027
Putamen	R	27	14	− 7	5.43	0.002^b^
Urge to pick						
Amygdala	R	21	− 7	− 16	3.57	0.034
Amygdala	L	-24	− 7	− 22	3.37	0.045
Tension						
Insula	R	36	− 22	5	5.26	0.004^b^
Insula	L	− 36	− 22	2	4.21	0.040
Amygdala	R	18	− 10	− 16	3.79	0.029
Amygdala	L	− 15	− 13	− 16	4.19	0.010
Putamen	R	30	5	2	8.28	0.001^b^
Putamen	L	− 24	11	2	4.82	0.006^b^

*H* hemisphere, *x,y,z* MNI-coordinates, t-value, *p(FWE)* p-value corrected for family-wise-error, *w* significant on the whole brain level, *b* significant with Bonferroni-correction

Correlations between ROI activation (contrast: skin irregularities - smooth skin) and questionnaire scores are depicted in Table 3. Picking type (MIDAS focused picking) was positively associated with amygdala activation. Additional correlations between symptom severity (SPS_R), self-disgust and ROI activation were present. However, these correlations were not statistically significant anymore after Bonferroni correction. None of the correlations for the control group reached statistical significance.

**Table 3:** Positive correlations between activation in regions of interest (ROIs) and questionnaire scores for patients

Contrast	ROI	H	X	Y	Z	t	p(FWE)
MIDAS_focused_picking	Amygdala	L	− 27	− 4	− 19	4.27	0.008^b^
MIDAS_focused_picking	Amygdala	R	33	− 1	− 16	3.57	0.033
SPS_R_impairment	Insula	R	39	17	2	4.33	0.023
SPS_R_ impairment	Putamen	R	30	8	− 1	3.75	0.048
SPS_R_ impairment	Motor cortex	R	6	8	62	4.10	0.040
SPS_R_total	Insula	R	39	20	2	3.99	0.044
SPS_R_total	Motor cortex	R	6	5	62	3.69	0.043
QASD_self-disgust	Putamen	L	− 27	− 13	− 7	3.89	0.037

*MIDAS* Milwaukee Inventory for the Dimensions of Adult Skin-Picking, *SPS_R* Skin Picking Scale revised, *QASD* Questionnaire for the Assessment of Self-Disgust, *H* hemisphere, *x,y,z* MNI-coordinates, t-value, *p(FWE)* p-value corrected for family-wise-error, *b* significant with Bonferroni-correction

#### PPI

The PPI analysis for the contrast skin irregularities - smooth skin showed enhanced connectivity in patients (relative to controls) between the left insula (seed) and the right putamen (MNI coordinate x,y,z: 33, − 16, − 4; t = 4.21, p = .006; CS = 118; see Fig. 1).

## Discussion

This fMRI study investigated neuronal correlates of state and trait disgust in patients with SPD in the context of symptom provocation. The participants were presented with images depicting skin imperfections and smooth skin. SPD patients reported elevated feelings of disgust when confronted with visual cues of dermatological problems, and showed increased activation of the left insula and left amygdala relative to controls. In addition, patients’ level of disgust experienced while viewing skin irregularities positively correlated with activation of the insula and the putamen.

Insula activation and joint insula-amygdala activation have been consistently identified during visual disgust elicitation in healthy controls as well as in different clinical samples, such as patients with obsessive–compulsive disorders (e.g., Schienle et al. 2002, 2005). The insula is closely connected with the amygdala, which has a paramount role in processing salience and affective significance of stimuli (LeDoux 2014). The insula holds a common representation of disgust observation, experience and imagination. More generally, the insula is a central hub for interoceptive and affective awareness (Grupe and Nitschke 2013). This region is involved in the perception of bodily experiences, such as touch, and itch. Lucas et al. (2015) identified a functional dissociation between posterior insula regions, which responded to actual touch, and the anterior insula, which was activated during both experienced and imagined touch. Insula activation, as well as the recruitment of the putamen played a role in the urge to scratch and the subsequent relief derived from scratching (Papoiu et al. 2013). This mechanism seems to be deeply rooted in humans’ evolutionary history. Itch is a typical symptom that occurs after contact with irritants. It can be a sign of skin infestation by parasites (e.g., scabies), or of infection. In order to get rid of these conditions, both the skin sensation, but also visual cues (dermatological irregularities such as bumps, pimples) trigger scratching and cleaning, as natural remedies.

The patients of the current study reported elevated state disgust with regard to skin imperfections, but also increased trait disgust (self-disgust and disgust sensitivity). Self-disgust is a personality trait that refers to an extreme dislike of oneself. This may include one’s own bodily features or actions. Self-disgust is essentially absent in healthy individuals, but has been identified as a symptom of specific mental disorders, such as borderline personality disorder, depression, and eating disorders (Ille et al. 2014; Schienle et al. 2014). The common feature of these disorders is devaluing the own person as ‘wrong’ or ‘ill’. Self-disgust scores of the patients were positively correlated with putamen activation. Classical investigations (Calder et al. 2000; Phillips et al. 1997) have already underlined that the insula-putamen system is involved in disgust processing across different sensory modalities (visual, auditory). Calder et al. (2000) studied a patient (NK) with circumscribed neural damage of these two regions. NK showed a largely selective deficit in recognizing and experiencing disgust. Further, abnormally high activations of the insula and the striatum (putamen, caudate nucleus) are implicated in OCD psychopathology, especially in those patients with contamination fears and compulsive hand washing (Schienle et al. 2005). In addition, the insula and the putamen showed enhanced coupling in the clinical group. Questionnaire scores reflecting symptom severity (SPS) positively correlated with insula and putamen activation in the SPD patients. However, these correlations were not statistically significant after Bonferroni correction.

Finally, SPD patients indicated problems in controlling their own disgust feelings as reflected by increased disgust sensitivity scores. This finding corresponds with models of SPD that emphasize emotion regulation difficulties as a crucial pathological mechanism in this disorder. The patients experience aversive emotional states and respond with picking which provides relief. The picking is therefore negatively reinforced (e.g., Snorrason et al. 2010). Future studies are needed to investigate the regulation capacity of SPD patients with regard to other basic emotions (e.g., anger, sadness). Then it can be decided if problems with disgust control are only one facet of a broader emotion regulation deficit in SPD, or if disgust has a more specific role.

We need to mention the following limitations of the current study. The total sample size of the current study was relatively small. However, the clinical group consisted of SPD patients with low comorbidity. None of the interviewed patients reported clinically relevant symptoms of OCD or trichotillomania. Only two of the patients were medicated with antidepressants, and the results did not change when these two participants were excluded from the analysis. This low rate of psychiatric comorbidity and medication helps to detect disorder-specific neuronal features, but also reduces the representativeness and the generalizability of the present findings.

The degree of motion in the SPD group was higher than in controls, and two patients had to be excluded due to excessive motion artifacts. This problem occurred despite measures for reducing motion like using foam pads and the detailed instruction to lay still in the scanner bore. We applied the sophisticated motion fingerprint approach (Wilke 2012) in order to explain further motion-related variance; this approach calculates the average cortical-distance for each individual and considers individual brain anatomy.

In summary, this study provides first evidence for altered disgust processing in SPD patients. Visual cues of dermatological problems induced elevated disgust and tension in the patients, which was associated with activation of a network comprising the insula, amygdala and the putamen. Future studies are needed in order to specifically investigate the ‘pathological grooming model’ of SPD. For example, different affective states (e.g., disgust, fear, anger, happiness) could be elicited in the patients and the degree of skin-picking that occurs during these affective episodes could be compared with each other. If disgust indeed plays a crucial role in skin picking, then this emotion should specifically increase disorder-relevant behavior.
